# Hydrophobin Bilayer as Water Impermeable Protein Membrane

**DOI:** 10.1021/acs.langmuir.3c01006

**Published:** 2023-09-19

**Authors:** Friederike Nolle, Leonhard J. Starke, Alessandra Griffo, Michael Lienemann, Karin Jacobs, Ralf Seemann, Jean-Baptiste Fleury, Jochen S. Hub, Hendrik Hähl

**Affiliations:** † Department of Experimental Physics, Saarland University, D-66123 Saarbrücken, Germany; ‡ Department of Theoretical Physics, Saarland University, D-66123 Saarbrücken, Germany; § Max Planck School, Matter to Life, Jahnstraße 29, 69120 Heidelberg, Germany; ∥ VTT Technical Research Centre of Finland Ltd., Espoo 02150, Finland; ⊥ Max Planck Institute for Medical Research Heidelberg, 69120 Heidelberg, Germany

## Abstract

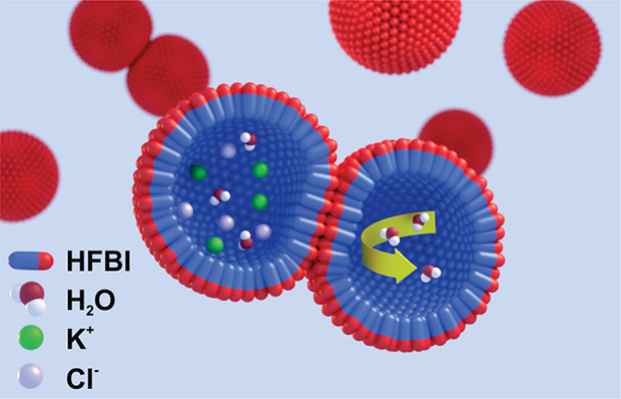

One of the most important properties of membranes is their permeability to water and other small molecules. A targeted change in permeability allows the passage of molecules to be controlled. Vesicles made of membranes with low water permeability are preferable for drug delivery, for example, because they are more stable and maintain the drug concentration inside. This study reports on the very low water permeability of pure protein membranes composed of a bilayer of the amphiphilic protein hydrophobin HFBI. Using a droplet interface bilayer setup, we demonstrate that HFBI bilayers are essentially impermeable to water. HFBI bilayers withstand far larger osmotic pressures than lipid membranes. Only by disturbing the packing of the proteins in the HFBI bilayer is a measurable water permeability induced. To investigate possible molecular mechanisms causing the near-zero permeability, we used all-atom molecular dynamics simulations of various HFBI bilayer models. The simulations suggest that the experimental HFBI bilayer permeability is compatible neither with a lateral honeycomb structure, as found for HFBI monolayers, nor with a residual oil layer within the bilayer or with a disordered lateral packing similar to the packing in lipid bilayers. These results suggest that the low permeabilities of HFBI and lipid bilayers rely on different mechanisms. With their extremely low but adaptable permeability and high stability, HFBI membranes could be used as an osmotic pressure-insensitive barrier in situations where lipid membranes fail such as desalination membranes.

## Introduction

All living cells are surrounded by lipid membranes, fulfilling similar tasks despite their different structures and chemical compositions. Membranes are responsible for the compartmentalization of living cells and control the selective transport between these compartments. The regulation of water permeability is particularly important for maintaining cell homeostasis,^1^ enabling the cell to respond to external influences, such as salt concentration or pH. Controlling water permeability is also relevant in the field of biomimetics for potential biotechnological and biomedical applications,^2−4^ for instance as nanocarriers, and has therefore been addressed by various experimental and theoretical studies.^5−7^ The lipid composition, as well as the content of proteins, channels, or nanoparticles, strongly influences the permeability, as has been shown in numerous experimental studies using planar lipid bilayers^8−13^ or liposomes.^14−17^ Artificial membranes with controlled permeability have been formed using several other building blocks besides lipids,^18^ such as fatty acids, synthetic lipids, (block co)polymers,^19−21^ engineered proteins, or peptides.^22,23^ Other possible building blocks for artificial membranes are amphiphilic proteins like the protein HFBI, which has been used to form pure protein membranes.^24^

HFBI is a globular protein from the family of class II hydrophobins produced by the filamentous fungus *Trichoderma reesei*.^25^ The molecular surface of HFBI contains a characteristic nonpolar region, also known as a hydrophobic patch, which enables the fungus to attach to hydrophobic solid surfaces, such as wood,^26^ and to expose the hydrophilic protein regions to the solvent.^27−29^ At the air–water interface, HFBI forms highly ordered honeycomb-like monolayers as shown experimentally by atomic force microscopy^30−33^ and cryogenic electron microscopy.^34^ These ordered monolayers have been described computationally by protein–protein docking and molecular dynamics simulations.^35^ HFBI monolayers at the air–water interface resemble phospholipids in their layer-forming properties: both HFBI and lipids orient their hydrophobic parts toward the air. Yet, it was shown that the formation of HFBI monolayers is mainly controlled by steric and electrostatic interactions and therefore differs from the adsorption kinetics of phospholipids and other surfactants.^36^

Hydrophobin boundary layers have been used for the coatings of surfaces,^37,38^ for immobilization of molecules and cells,^39,40^ or for therapeutic applications such as drug delivery.^41,42^ Maiolo et al.^42^ encapsulated gold nanoparticles in a hydrophobin monolayer shell, thereby preventing the premature release of drugs and allowing a concentrated drug release at the target site *in vivo*.

Joining two hydrophobin boundary layers leads to the formation of stable protein double layers.^24,43,44^ By contact of two HFBI layers with their hydrophobic sides, a bilayer membrane can be formed between two aqueous compartments, similar to black lipid membranes. Such HFBI bilayers have previously been formed before in a microfluidic setup allowing both optical access and electrophysiological measurements.^24,43^ Thus, properties such as adhesion between the bilayer sheets, bilayer thickness, and ion transport across the bilayer have already been studied. In addition, these HFBI bilayers exhibit high stability and exceptional resistance to lateral stress, which also facilitated the formation of vesicles from these bilayers.^24^ Yet, the molecular structure and water permeability of these HBFI bilayers are still unknown. Knowledge of the latter would, however, facilitate their use in medical applications such as biosensing, biomimetics, and vesicle-based drug delivery.

In this study, planar HFBI bilayers were generated at the contact site of two micelles. These HFBI droplet interface bilayers were used to determine the water permeability of the protein bilayers. The water permeability value was found to be extremely low. The aim of this work is to explore and explain this low water permeability in experiments and molecular dynamics (MD) simulations and to look for ways to disrupt the order of the protein membrane in order to control the water permeability.

## Results and Discussion

### Volume Change of Droplet Pairs Due to Osmotic Gradient

**Figure 1:**
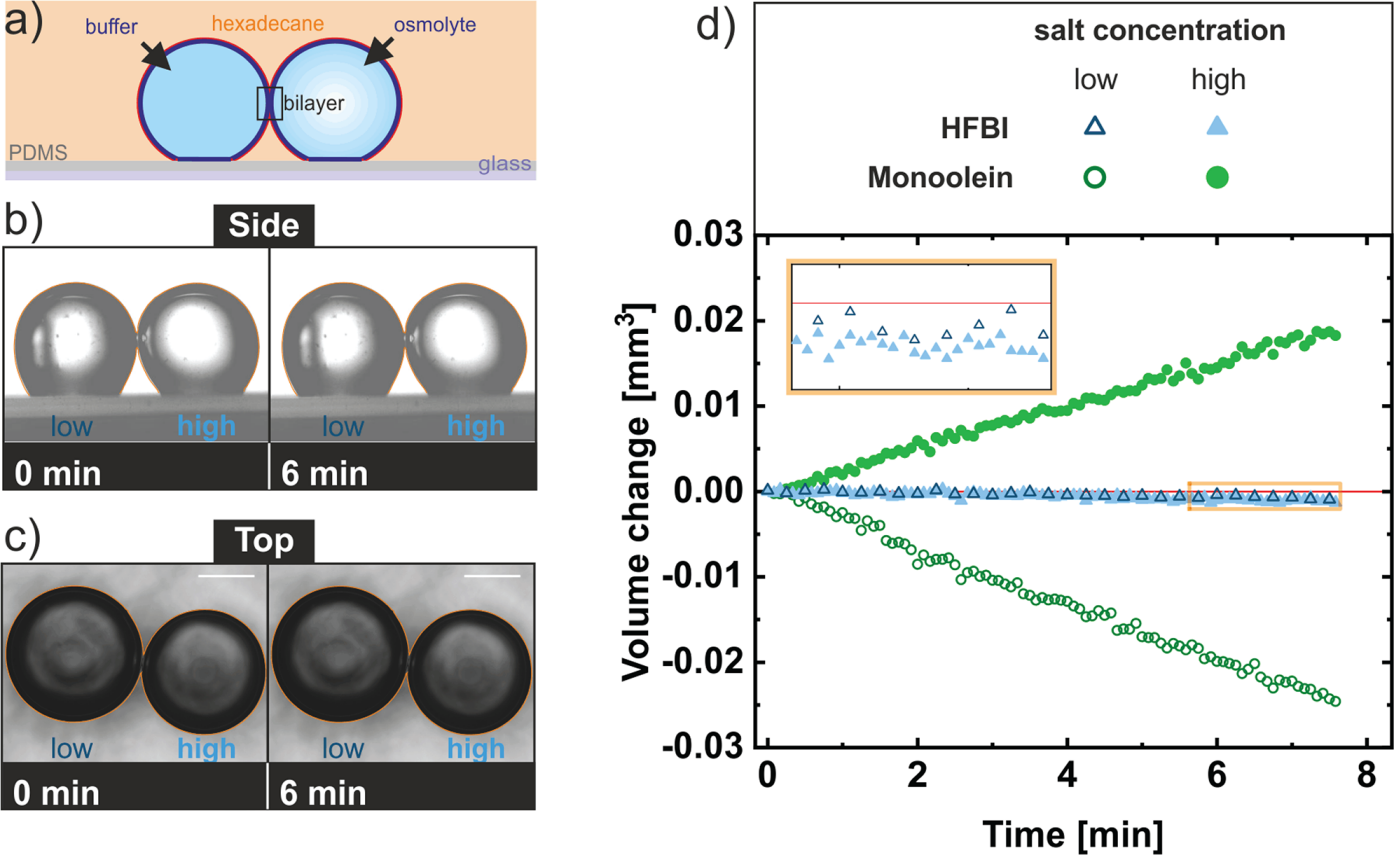
Droplet interface bilayer (DIB) experiments. (a) Sketch from the side of the experimental setup of two HFBI-coated buffer droplet pairs of different salt concentrations on a PDMS-covered glass substrate. (b, c) Side and top views of two HFBI-coated buffer droplets brought into contact and forming a DIB. The osmotic concentration difference of the two droplets was 1.717 osmol/L. The water permeability was measured by observing the volume changes of the individual droplets over time. The contour of the droplet pairs (orange) at the beginning of the measurements was copied and pasted into the image taken at 6 min. The scale bar indicates a size of 500 μm. (d) Volume change of droplets over time with pairs of monoolein (green circles) and HFBI (blue triangles) coated droplets. In both measurements, the osmotic concentration difference between the two droplets in contact was 0.259 osmol/L. The inset shows an enlargement of the volume change of the HFBI-coated droplet pair for the last 2 min. The red line indicates zero volume change.

By bringing two buffer droplets of different salinities in hexadecane surrounded by protein monolayers into close contact, a droplet interface bilayer (DIB) is formed (Figure 1a). An osmotic pressure caused by the difference in salinity leads to a flux of water from the droplet with lower salinity to the one with higher salinity, if the bilayer is water-permeable. Figures 1b and 1c show an HFBI-coated droplet pair with an osmotic concentration difference of 1.717 osmol/L immediately after contact (left) and 6 min later (right). No volume changes were optical discernible. For comparison and closer examination, we recorded the volume change of droplet pairs covered with HFBI in hexadecane (cf. blue data in Figure 1d) and droplet pairs with monoolein in squalene (cf. green data in Figure 1d) with an osmotic concentration difference of 0.259 osmol/L. (Squalene was chosen for the oil phase due to the improved stability of the bilayer with respect to bilayers formed in hexadecane.) Compared to bilayers formed by other lipids, monoolein forms bilayers with a relatively low permeability.^8,13,45^ Still, no discernible volume change was observed for HFBI droplet pairs in comparison to that for monoolein droplet pairs. To confirm that no remaining oil in the bilayer was the reason for this water impermeability, we used BODIPY as an oil tracer.^46^ No oil was detected in the bilayer with this method (Figure S1). For these pure protein bilayers, it was also previously shown by capacitance measurements that essentially no hexadecane remains between the layers.^24^

A small volume decrease (about 0.001 mm^3^) was observed in both HFBI-covered droplets (see the inset of Figure 1d). This water loss was characterized in single-droplet measurements as diffusion of water into hexadecane with a diffusion coefficient in the range (4–6) × 10^3^ μm^2^/s (Table S1). Because this diffusion coefficient is in the same range as for simple water droplets in hexaxdecane, this result implies that the water passes through the hydrophobin monolayer without any additional hindrance. Hence, it was necessary to account for the water diffusion into hexadecane by correcting the data before calculating the membrane permeability. For this purpose, the total volume loss of a pair of droplets was determined, and this loss was added to the volume of the individual droplets, according to the ratio of their surface areas. Thus, in the corrected data, the influx of one droplet was equal to the outflow of the second droplet. After this correction, the data for the HFBI membrane suggest that there is no water exchange between the droplets, so no values for water permeability were calculated. In contrast, there was a clear difference in volume change for the monoolein droplet pairs in squalene; however, the volume loss (∼0.025 mm^3^) of the droplet with the low salinity was larger than the volume gain (∼0.020 mm^3^) of the droplet with high salinity. This indicated that there was also an overall volume loss into squalene. The permeability obtained from the corrected data for the monoolein membranes was 56 ± 1 μm/s at 30 °C and thus in the range reported in other studies (58 ± 3 μm/s at 25 °C in squalene).^8^

These results show that under the tested experimental conditions for HFBI membranes, in contrast to monoolein membranes, (i) the flux through the HFBI membrane is smaller than the flux into the surrounding hexadecane and (ii) the chosen time interval and osmotic concentration difference are insufficient to determine a water permeability for pure protein membranes. Therefore, the osmotic concentration difference as well as the time interval was largely increased in the experiments described below.

### Permeability of Pure HFBI Membranes

For more precise measurements of the water permeability of the HFBI membranes, the osmotic concentration difference between the droplets was increased to 1.717 osmol/L and the observation time was extended to 30 min after the initial contact. HFBI membranes are capable of resisting this high osmotic pressure and are stable over long periods of time, up to several days (in contrast to lipid membranes, which cannot form stable membranes under these osmotic conditions).

**Figure 2:**
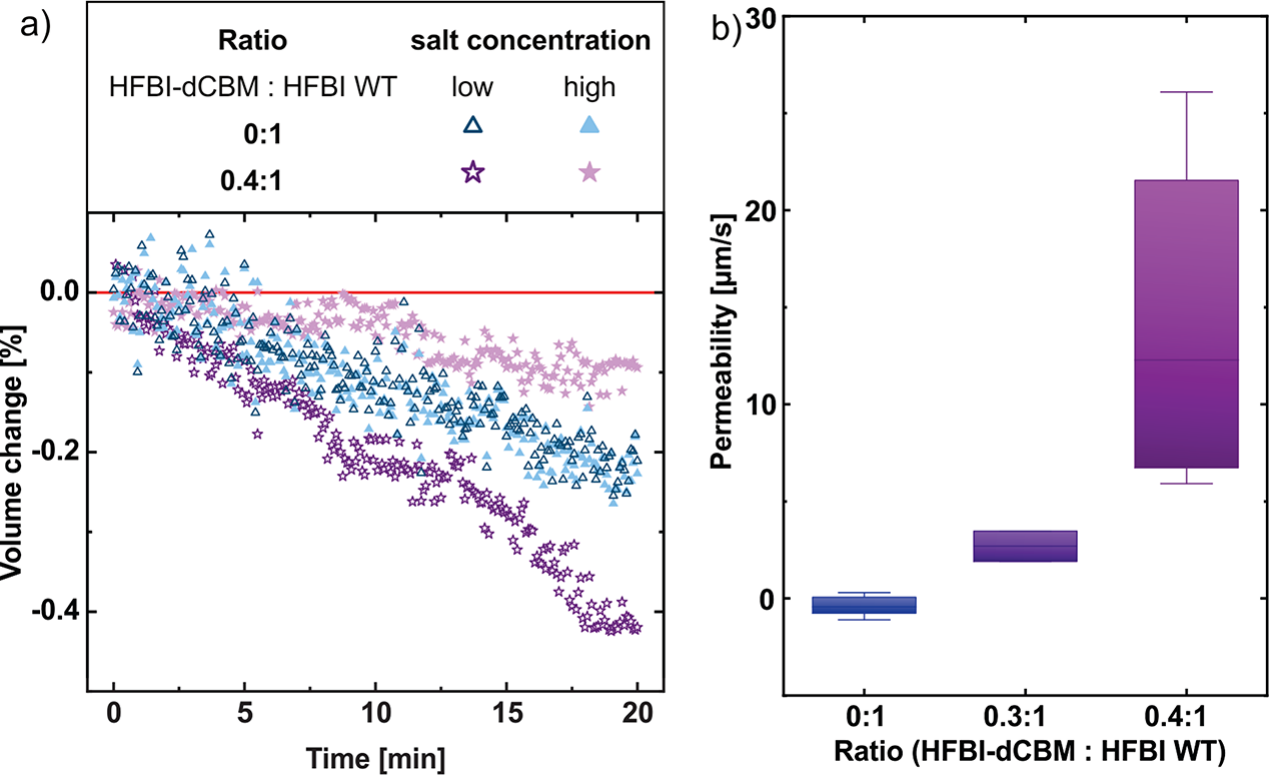
(a) Volume change in percent of droplets, with initial size of *V*_0_ = 0.7–0.9 mm^3^, over time with droplet pairs having a HFBI-dCBM:HFBI WT ratio of 0:1 (blue triangles) and 0.4:1 (purple stars). (b) Box and whisker plots (min-to-max) of the mean permeability values of several HFBI membranes in the presence of the mutant HFBI-dCBM in different weight ratios. Osmotic concentration difference: 1.717 osmol/L for pure HFBI membranes and 0.086 osmol/L for HFBI-dCBM:HFBI mixtures. Temperature: 30 °C.

However, the results of the second series of experiments (Figure 2a, blue triangles) were similar to the results of the previous ones: both droplets shrink in the same range of order. It is noteworthy that for the given contact area and high osmotic pressure, a permeability of only about 6 μm/s would be sufficient to compensate for the diffusion of water into hexadecane, i.e., to keep the volume of the droplet with the high salt concentration constant. Thus, even without further analysis, the measurements show that the water permeability of the HFBI membrane is well below 6 μm/s.

After volume correction, no volume transfer from the low salinity droplet to the high salinity droplet is detectable. Taking into account the average size of the droplets and their common contact area, as well as the error in volume determination (8 × 10^–4^ mm^3^), permeabilities with an accuracy of 1 μm/s are accessible. With this accuracy of the current setup, the observed water permeability of HFBI membranes is indistinguishable from zero (see the Methods section: Error analysis in the determination of experimental membrane water permeability). These results are surprising given that water passes through HFBI monolayers into the oil just as unimpeded as through uncoated water droplets (Table S1). The permeability through the HFBI monolayers can be understood based on the known honeycomb structure at the air–water interface.^30,32^ Thus, for the membrane we hypothesize a rearrangement of proteins into a much more densely packed arrangement.

To test this hypothesis, the structure of the monolayer (composed exclusively of wild-type HFBI) was disrupted by inserting the bulky HFBI fusion protein HFBI-dCBM, which is composed of two cellulose-binding domains bound to the wild-type HFBI domain.^47^ Weight ratios of 0.3:1 and 0.4:1 of HFBI-dCBM:HFBI WT were used in the bulk concentrations of the droplets. The osmotic concentration difference 0.086 osmol/L was chosen at a temperature of 30 °C. The results of these experiments are displayed in Figure 2b, and the experimental data corrected for the water loss into the oil are shown in Figure S2. In contrast to the experiments with the pure HFBI membrane, clear differences in the volume change were observed for both mixing ratios tested. Volume decrease occurred still in all droplets, although the volume loss was significantly less in the droplets with high salt concentration than in the droplets with low salt concentration (Figure 2a, purple stars). This results in clearly detectable positive permeability values of around 3 μm/s for the ratio of 0.3:1 (HFBI-dCBM:HFBI) and up to 27 μm/s for the ratio of 0.4:1 (Figure 2b). For the latter ratio a higher variation of permeability values was observed, yet always higher than for the ratio of 0.3:1. Apparently, the addition of HFBI-dCBM resulted in a detectable flux between two droplets with an osmotic gradient. This demonstrates that the impermeability of the pure HFBI WT membrane to water and ions is the reason no significant volume change occurs across the bilayer between the droplet pair.

To conclude, within the tested regime of osmotic gradients of 0.259–1.717 osmol/L, which was generated by the addition of either KCl or NaCl (see the Supporting Information), pure HFBI membranes are impermeable to water within our measurement accuracy of about 1 μm/s. The permeability is much lower compared to water permeability values estimated by fluorescence self-quenching in liposomes composed of palmitoyloleoylglycerophosphocholine (POPC) and cholesterol, which have water permeabilities between 72 ± 18 μm/s (pure POPC) and 13 ± 5 μm/s (POPC:cholesterol ratio 60:40).^14^ The permeability of HFBI is even lower than the permeability of densely packed sphingomyelin:cholesterol (60:40) membranes of 2.2 ± 0.4 μm/s, although their permeabilities may be of a similar order.^14^ A measurable water permeability of HFBI membranes can be achieved by adding a bulky HFBI fusion protein, but at the tested ratios it is still low compared to monoolein and other lipid membranes.

To better understand the effect of HFBI ordering in the membrane on the water permeability, MD simulations were initiated. MD simulations were already successfully used previously to study water permeation across lipid membranes,^48^ aquaporins,^49,50^ and other nanopores.^51^ Furthermore, MD simulations were used to study class II hydrophobins within solution^52^ and at interfaces.^35,53,54^

### MD Simulations of HFBI Monolayers at the Air–Water Interface Are Stable

**Figure 3:**
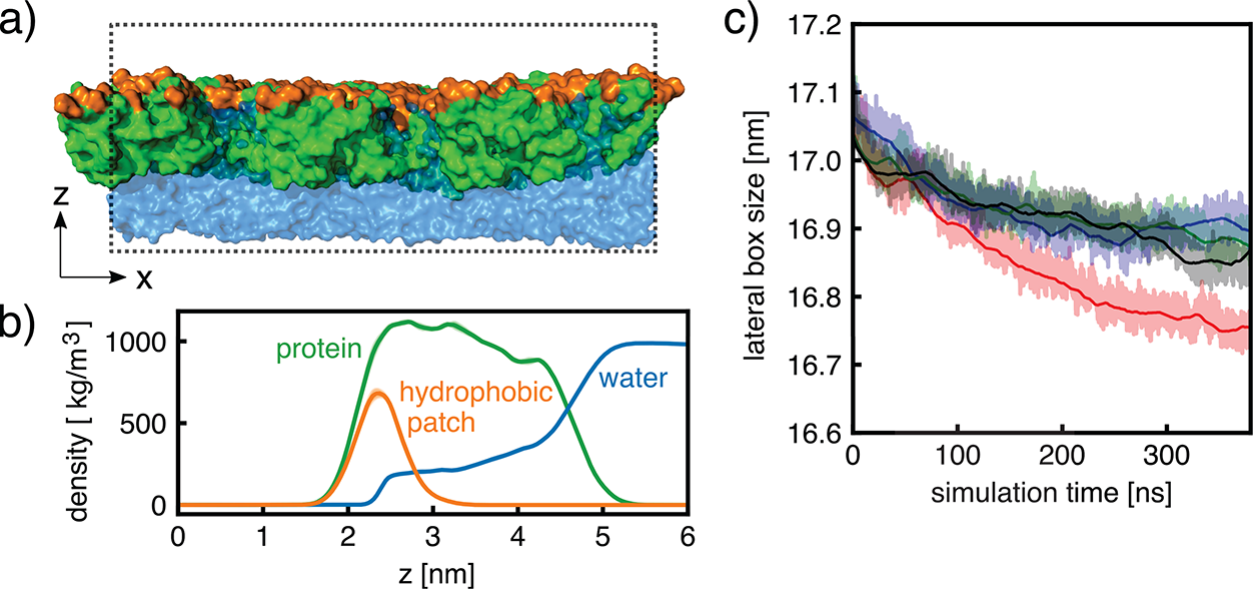
HFBI monolayer simulations. (a) Side view of simulation system shown in surface representation composed of proteins (green) with hydrophobic patches (orange) and water (blue). The simulation box is shown as a dotted black line. (b) Mass density along the membrane normal averaged over four independent simulations. (c) First lateral box dimension versus time taken from four independent simulations (shaded areas). Lines show running averages to guide the eye.

To obtain molecular models of the HFBI monolayers and bilayers and to study water permeation over HFBI bilayers in atomic detail, we used all-atom MD simulations. As starting conformations, we used the monolayer arrangements obtained via protein–protein docking by Magarkar et al.,^35^ which were designed to reproduce the honeycomb structure observed in HFBI monolayers.^30^ The authors reported two different unit cell models termed HFBI-α and HFBI-β (Figure S4). The two models exhibit an overall similar monolayer packing yet with distinct protein–protein interfaces, as is evident from the relative orientation of the α-helices within the unit cells (Figure S4). Two salt bridges were found in the HFBI-β unit cell, with a dominating one between Asp^30^ and Lys^32^ suggesting a higher stability compared to the HFBI-α unit cell. To reveal the interactions of water with the HFBI monolayer, we performed simulations of the HFBI monolayer based on the HFBI-β unit cell at the air–water interface at constant pressure (Figure 3a). Over four independent 400 ns simulations, the monolayer was largely stable, exhibiting only a minor decrease of the lateral simulation box size of ∼1 to 2% reflecting a minor tightening of the protein–protein interfaces (Figure 3c). Analysis of density profiles along the membrane normal reveals a large overlap between the protein and water densities, demonstrating that the HFBI monolayer is largely hydrated (Figure 3b). Notably, the water density extends partly into the layer of hydrophobic patches, rationalized by the polar side chain of Gln^65^ and by polar backbone atoms in the hydrophobic patch. Overall, the monolayer simulations based on the unit cells proposed by Magarkar et al.^35^ are compatible with the experimentally observed honeycomb structure of the monolayer. A similar simulation of the HFBI monolayer at a hexadecane/water interface was performed in addition, again showing that the honeycomb structure is stable (Supporting Information).

### MD Simulations of Bilayers with Honeycomb Structure Exhibit Massive Water Leakage

**Figure 4:**
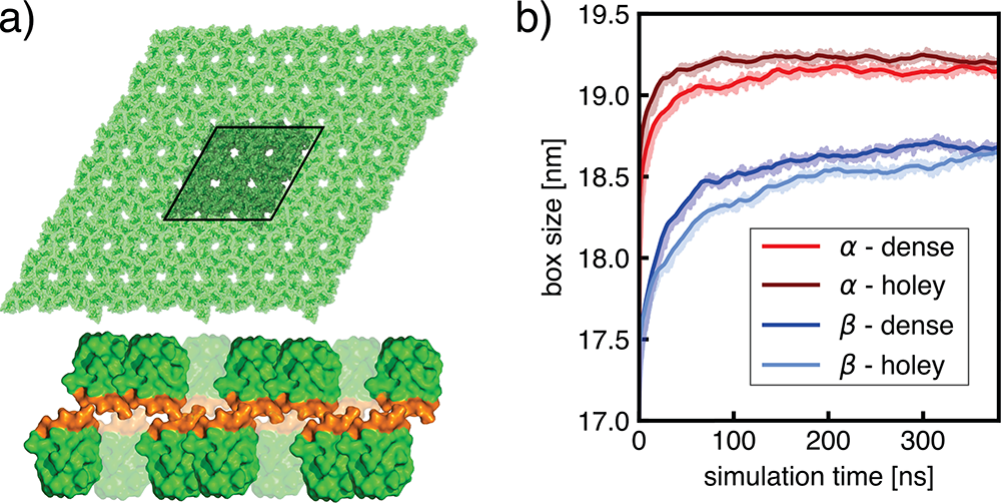
(a) Top: top view of the simulation setup of HFBI with a honeycomb structure. One simulation cell is colored dark green. Light green regions depict periodic images of the cell. Bottom: schematic view of the “dense” overlay of two laterally displaced monolayers with cavities spanning only one leaflet. (b) Example of expansion of the first lateral box dimension during simulations based on the HFBI-α (red) and HFBI-β (blue) unit cells,^35^ composed of either the “dense” or the “holey” membrane model (see legend).

**Figure 5:**
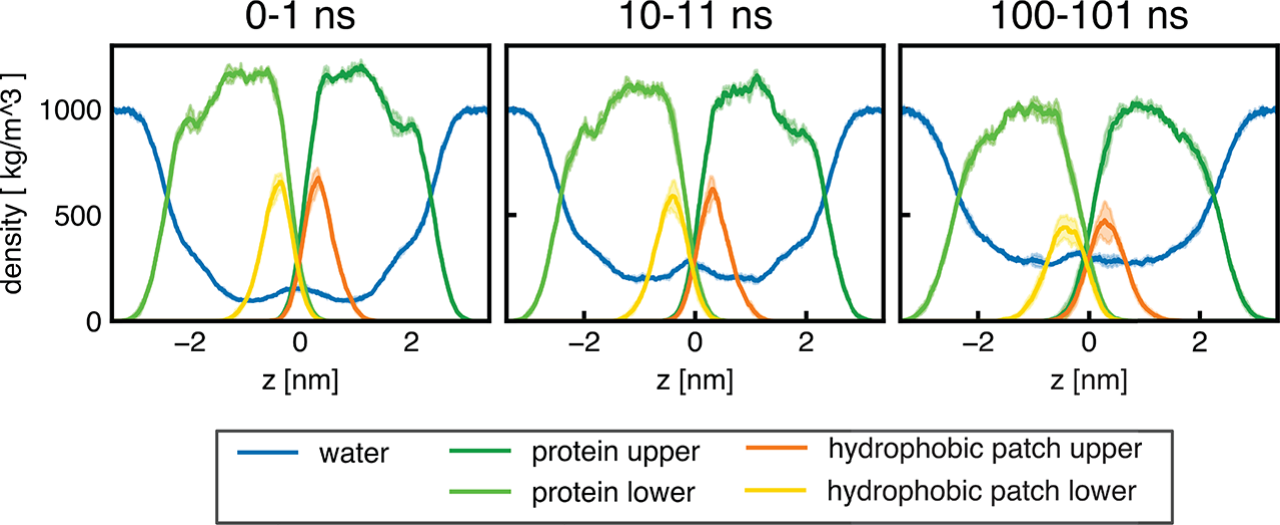
Mass densities of HFBI (green), hydrophobic patches (orange), and water (blue) for the “dense” HFBI bilayer based on the HFBI-β unit cell taken from different time intervals 0–1 ns (left), 10–11 ns (middle), and 100–101 ns (right). Contributions from the upper and lower leaflet are plotted in different shades (see legend).

Next, we used MD simulations to test whether the honeycomb structure observed in monolayers is compatible with the low water permeability of HFBI membranes observed in our DIB experiments. To this end, we overlaid two monolayers with the hydrophobic patches facing each other based on two different lateral arrangements: the cavities of the honeycomb structure were either aligned (i) between the two leaflets, such that large continuous transmembrane pores were formed, denoted “holey membrane”, (ii) or the cavities were laterally displaced such that the cavities spanned only one leaflet, denoted “dense membrane” (Figure 4a, bottom). Each lateral arrangement was built with either the α or the β model by Magarkar et al.^35^ Within 400 ns of simulation, we observed massive water permeation across the transmembrane pores of the holey structure. However, major water leakage was also observed in the dense structure with laterally displaced cavities. By counting water permeation events over the bilayer, we obtained permeabilities in the range 400–24000 μm/s, in stark contrast to the experimental results (Table 1). In addition, the simulation box expanded laterally by 5–10% over the simulation time, resulting in a destabilization of the lattice structure due to translational movement of the individual proteins (Figure 4b), suggesting that the initial membrane configuration was not optimal. To exclude that the observed water leakage is a force field related artifact, we performed additional simulations with the OPLSaa^55^ and AMBER99SB^56^ force fields as well as simulations using the CHARMM36m force field in combination with the OPC water model.^57^ All simulations showed the same qualitative behavior of rapid water intrusion, indicating that the observed effect is not a force field-specific artifact (Figure S6). Analysis of density profiles of protein and water confirms that water increasingly penetrates the bilayer within the first 100 ns of simulation such that the water molecules are located at the hydrophobic patch at a reduced density of ∼400 kg/m^3^ (Figure 5). The penetration of water is not surprising considering that water was in contact with the hydrophobic patches even in the monolayer simulations (Figure 3). These findings suggest that the mere presence of the thin hydrophobic patch is insufficient to exclude water permeation across the HFBI bilayer. Additional simulations of honeycomb bilayers including a thin film of hexadecane between the monolayers were performed (Supporting Information). These simulations confirmed the experimental observation that no residual oil film remains, which could prohibit water permeation.

**Table 1:** Water Permeability of HFBI Bilayers Based on HFBI-α and -β Unit Cells with Dense or Holey Lateral Arrangements

system	H_2_O permeability [μm/s]
HFBI-α, dense	10000 ± 2000
HFBI-α, holey	23920 ± 50
HFBI-β, dense	3700 ± 200
HFBI-β, holey	9800 ± 500
disordered HFBI	13000 ± 8200
experiments	0 ± 1

### Exceptionally Tight Protein–Protein Interactions Are Required to Explain Low Water Permeability

**Figure 6:**
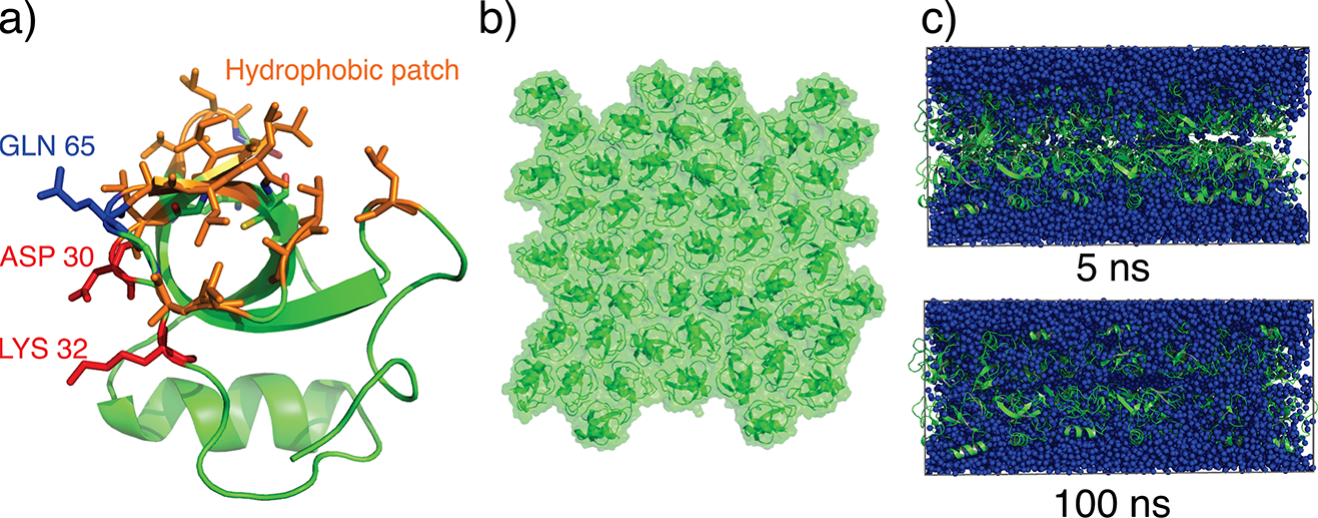
(a) Scheme of the HFBI structure. Secondary structure is shown in green and the hydrophobic patch in orange including the side chains. In addition, the charged side residues Asp^30^ and Lys^32^ and the polar side chain of Gln^65^ are highlighted in red and blue, respectively. (b) Graphical representation of a dense disordered HFBI monolayer (top view) after compression procedure. (c) Snapshots (side view) from two different time points of a HFBI bilayer simulation build of two monolayers as represented in (b). Water is shown in blue.

Lipid bilayers represent a two-dimensional fluid in which the lipid molecules are relatively loosely packed. Water permeation in those bilayers is suppressed owing to the nanometer-sized hydrophobic core, which disfavors the partitioning of polar water molecules. We used MD simulations to test whether a laterally dense, irregular hydrophobin packing, similar to the lipid packing in lipid bilayers, would be sufficient to exclude water permeation. In free MD simulations, however, hydrophobins did not form laterally tight packing within accessible simulation times as a consequence of long-living protein–protein contacts. Hence, we devised a multistep protocol based on coarse-grained (CG) models and lateral compression simulations using large lateral pressures and high temperatures, followed by backmapping of the CG to atomistic models (see the Supporting Information). Such an artificial protocol does not give insight into the physical self-assembly or reorganization process but provides a structural model of a densely packed hydrophobin bilayer, as evident from the continuous (transparent) molecular surface rendered in Figure 6b. This model was used as a starting point for further simulations.

However, as illustrated in Figure 6c, even the densely packed hydrophobin bilayer was penetrated by water within short simulation times, irrespective of the equilibrium protocol (Supporting Information). Visual inspection of the simulations showed that the water penetrated primarily at contact sites with Asp^30^ and Lys^32^ residues, rationalized by the high water affinity of these ionic residues. Furthermore, other polar residues at the protein–protein interfaces became rapidly hydrated, such as Gln^16^, Thr^20^, Gln^69^, Thr^70^, and Asn^72^. These water protrusions further caused the loss of some protein–protein contacts and led to a lateral expansion of the bilayer by approximately 7–10%. After 100 ns of simulation, the bilayer was largely hydrated. The permeability was approximately 1 cm/s, which was incompatible with our experimental data (Table 1). These findings demonstrate that overall tight but irregular lateral packing of HFBI monomers, similar to the lipid packing according to the two-dimensional fluid mosaic model of lipid membranes, is insufficient to rationalize the experimental water permeabilities. Instead, we propose that well-defined and enormously tight protein–protein interactions are required to form a stable densely packed bilayer. The required reorganization process not only needs to introduce a few specific bonds that link the proteins together (as was the case for the unit cells at the air–water interface) but also needs to form a near-perfect interlocked protein–protein interface to act as an effective barrier against water penetration. Such a reorganization process occurs most likely at time scales inaccessible to MD simulations and thus cannot be resolved within this study.

## Conclusions

Using a DIB setup, we showed that pure HFBI membranes are impermeable to water with an experimental accuracy of ∼1 μm/s. Hence, HFBI membranes provide novel biocompatible membranes with exceptionally low water permeability and high stability.

We used MD simulations to test several structural hypotheses for rationalizing the low HFBI permeability. These hypotheses were based on low-resolution structural data of HFBI monolayers, which revealed a monolayer honeycomb structure,^30−33^ and on the expectation that physiochemical mechanisms applying to lipid membranes might likewise apply to HFBI membranes. The simulations suggest that the large cavities in the honeycomb structure are incompatible with the experimentally found low HFBI membrane permeability. The cavities of the honeycomb structured HFBI monolayer became rapidly hydrated, leading to major leakage of the membranes. In addition, our simulations together with analysis of the HFBI structures excluded the possibility that the low water permeability is caused by an extended hydrophobic core as present in lipid bilayers (thickness of 2–3 nm) because the hydrophobic patch of HFBI forming the central layer is only a few angstroms thick. The hydrophobicity of the HFBI bilayer core is further reduced by the presence of polar atoms of the protein backbone and by the Gln^65^ side chain. The presence of an only angstrom thick hydrophobic layer is reflected by the extended hydration of hydrophobin monolayers at the water–air interface (Figure 3), where the water density reaches up to the layer of the hydrophobic patch. The simulations revealed water leakage even for dense yet irregular lateral HFBI packing (Figure 6), similar to the irregular lateral packing in lipid bilayers according to the fluid mosaic model. Hence, the presence of the moderately apolar angstrom thick layer is far from sufficient to explain the low permeability of the HFBI membrane.

To experimentally demonstrate that the low permeability is indeed an intrinsic property of the HFBI membrane, we used an HFBI variant with two cellulose–binding domains. The additional domains led to an increased water permeability comparable to the permeability in monoolein, likely by precluding the formation of a tight lateral packing of HFBI monomers. Furthermore, we excluded experimentally and by simulations the possibility that the low water permeability is caused by a residual film of oil between the HFBI leaflets.

Based on these results, we propose that the formation of a HFBI membrane from two HFBI monolayers in a honeycomb structure triggers a lateral rearrangement of HFBI monolayers, leading to an exceptionally dense packing with well-defined, stable protein–protein interfaces. We anticipate that such interfaces are at least as tight as those found in sphingomyelin:cholesterol membranes, which exhibit a comparable low water permeability. Owing to long-lasting protein–protein interactions, these lateral rearrangements likely occur on long time scales that are currently inaccessible to MD simulations. Hence, to rationalize the experimentally found low water permeability by atomic models and by MD simulations, it will be critical to obtain atomic-level structural information in future studies, for instance, via cryo-electron microscopy or NMR spectroscopy, which may help in defining the protein–protein interfaces and, thereby, guide future simulations.

To conclude, we found that HFBI membranes exhibit exceptionally low water permeability and are capable of withstanding high osmotic pressures. These features are in contrast to lipid membranes, hence opening new options for using HFBI membranes as robust biomimetic membranes with preselected properties, for instance by incorporation of functional channels.^24^ This study lays the foundation for developing hydrophobin membranes toward a biocompatible platform for biophysical or biotechnological applications.

## Methods

### HFBI

HFBI is a class II hydrophobin naturally produced by the filamentous fungus *Trichoderma reesei*. It is highly amphiphilic with a hydrophobic patch and a kind of “hydrophilic pole”.^43^ Due to its compact size (ca. 7.5 kDa) and the presence of four disulfide bridges, HFBI is an exceptionally stable protein. In addition to the wild type, the HFBI variant HFBI-dCBM (ca. 18.5 kDa) was used.^58^ In this non-natural protein, two cellulose binding domains are bound to HFBI via a 24 amino acid long, unstructured linker (11 kDa).^47^ The lyophilized HFBI proteins used in this work were produced and purified at VTT (Espoo, Finland), as described in Paananen et al.^59^

Lyophilized HFBI was dissolved in 10 mM acetate buffer (pH ∼5) at a concentration of 100 μM. This stock solution was diluted to a concentration of 4 μM for further use. At this concentration, the droplets (radii: 0.52–0.62 mm) contain almost double the amount of proteins needed for full surface coverage of 0.45 μmol/m^2^.^36^ The dilution was performed by the addition of either a 10 mM acetate buffer with an ionic strength of 6 mM for the droplets with low salt concentrations or the same buffer supplemented with KCl to obtain an ionic strength of 954 mM for the droplets with high salt concentration. Therefore, a pair of droplets with an osmotic concentration difference of 1.717 or 0.259 osmol/L and for the HFBI-dCBM droplets of 0.086 osmol/L (osmotic coefficient: ϕ = 0.9; number of ions KCl dissociates: *n* = 2) were produced. All of the protein solutions were stored at 4 °C and sonicated prior to usage.

### Monoolein Solutions

Monoolein (Sigma-Aldrich, M7765, ≥99%) was dissolved in squalene (Sigma-Aldrich, S3626, ≥98%) (10 mg/mL) at 45 °C for around 20 min. The water droplets in the measurements with monoolein were prepared with an osmotic concentration difference of 0.259 osmol/L.

### Measurement Setup

All experimental measurements with HFBI were performed in *n*-hexadecane (Sigma-Aldrich, 8.20633, ≥99%) and all measurements with monoolein in squalene (Sigma-Aldrich, S3626, ≥98%). Hexadecane was chosen as the surrounding medium because it has already been shown that a HFBI bilayer can be produced in this oily phase.^43^ Squalene was used instead of hexadecane as a surrounding medium for monoolein, as no stable bilayers could be formed with monoolein in hexadecane. In order to avoid hexadecane crystallization, a temperature of 30 °C was chosen for all measurements, which is above the melting point of hexadecane (18.18 °C). To ensure a small contact area between the introduced droplets and the bottom, but also to prevent complete spreading of the droplets on the bottom, glass Petri dishes were coated with PDMS (Sylgard 184, Dow Corning) in order to increase their hydrophobicity (see Figure 1a).

### Bilayer Formation: Droplet Interface Bilayers

The bilayer formation for the water permeability measurements were oriented on the so-called “DIB” method (droplet interface bilayer^60,61^), which was previously used to calculate the permeability of lipid bilayers.^8,9,13^ Therefore, small droplets (radii: 0.52–0.62 mm) of aqueous solution were formed in oil. After a relaxation time of at least 30 min, during which the molecules were allowed to adsorb at the interface of the droplets and form a dense monolayer,^36^ two droplets with different osmotic concentrations were brought into contact with a metal needle to form a bilayer. The contact area formed in this way has already been shown to be a protein bilayer and is impermeable to ions, as shown by measurements of the layer thickness and voltage-clamped membrane current.^24^

The droplets were imaged by a top view light microscope (Leica DMI 2700M equipped with camera Leica MC170 HD), and their volume change was recorded for up to 30 min. The droplet volumes and bilayer areas were estimated from the recorded images. For determination of the droplets’ cross-sectional area from the images, a MATLAB program developed in-house was used. The grayscale images were first segmented in the foreground and background by thresholding. These foreground and background markers were then used to support edge detection and a final watershed segmentation to mark the droplets and the dividing line. The cross-sectional droplet area and the length of the dividing line were then used to calculate the volume of the droplets and the area of the bilayer, respectively, assuming a spherical shape of the droplets and a circular contact area.

Furthermore, a lateral control measurement by an optical contact angle instrument (OCA 25, Data Physics Instruments GmbH) was performed during which no flattening of the droplets could be observed (see Figure 1b), in contrast to hydrophobin droplets in air.^32^

Measurement noise in the determination of the cross-sectional area results in an error in the determination of the equivalent radius of 0.1 pixel, which corresponds to a measurement accuracy in the volume of 8 × 10^–4^ mm^3^ for the considered droplet volume. Using typical values (initial volume, osmotic concentration, and contact area), the permeability accuracy is 9 μm/s for the low salt concentration and 1 μm/s for the increased salt concentration. The permeability accuracy improves with higher osmotic concentration difference due to an increased volume change over time.

### Setup of Atomistic Molecular Dynamics (MD) Simulations of HFBI Monolayers at the Air–Water Interface

MD simulations were set up and performed with the GROMACS software.^62^ The HFBI structure files were retrieved from the Protein Data Bank (entry 2fz6).^63^ Crystal water and zinc ions were removed from the structure. The HFBI monolayers were constructed from one of the hexameric unit cells proposed by Magarkar et al.,^35^ denoted HFBI-β (Figure S4b). For the HFBI simulations at the air–water interface, nine of such unit cells were assembled in a 3 × 3 shape into a hexagonal lattice structure and centered in a triclinic box with dimensions 17.1 nm × 17.1 nm × 8.5 nm. Water was added in a slab between 2.1 nm < *z* < 6.0 nm to generate the air–water interface. The water layer was stabilized by using flat-bottom positional restraints with a force constant of 200 kJ/(mol nm^2^). The system contained 54 protein monomers and ≈20000 water molecules, which summed up to ≈120000 atoms. The CHARMM36m^64^ force field together with the TIP3P water model^65^ was used for the all-atomistic simulations. Virtual hydrogen site construction was enabled throughout, allowing a time step of 4 fs.^66^ The temperature was kept constant using the velocity-rescale thermostat^67^ at 300 K. Pressure coupling at 1 bar was applied using the Berendsen barostat in the lateral (*xy*) membrane direction.^68^ Electrostatic interactions were calculated using the particle-mesh Ewald method.^69^ Lennard-Jones interactions were cut off at 1.0 nm. Water molecules were constrained with SETTLE.^70^ All other bonds were constrained with LINCS.^71^ An initial constant volume (NVT) equilibration simulation over 50 ns was performed, followed by four independent 400 ns simulations. Mass density profiles along the membrane normal were calculated using the gromacs tool “gmx density”.

### Setup of Honeycomb Bilayer Simulations

HFBI monolayers were constructed as described above by using both proposed monolayer structures HFBI-α and HFBI-β (Figure S4). For each monolayer, nine of such unit cells were assembled in a 3 × 3 shape into a hexagonal lattice structure and centered in a triclinic box with dimensions 16.41 × 16.41 × 10 nm^3^ for HFBI-α and 17.16 × 17.16 × 10 nm^3^ for HFBI-β. Two copies of a monolayer were placed on top of each other such that the hydrophobic patches pointed inward. The upper and the lower monolayer were placed in two lateral arrangements: For the holey bilayer, the holes of the honeycomb structure were placed on top of each other, such that large transmembrane cavities were formed. In addition, for a dense bilayer the holes in the upper layer were laterally displaced relative to the lower bilayer, such that the holes in one leaflet were covered by proteins of the other leaflet. The simulation box was filled with explicit water molecules and with Na^+^ and Cl^–^ ions to reach a concentration of 0.1 mol/L. The system contained 108 protein monomers, ∼30000 water molecules, and ions, which summed up to ∼200000 atoms. All simulation parameters were identical with the honeycomb monolayer simulations at the interface, except that the pressure coupling was also applied separately along the membrane normal (*z*) direction and no positional restraints were used. For each setup, three independent simulations of 400 ns each were carried out. Mass density profiles were calculated as stated above.

### Setup of Disordered and Laterally Compressed Bilayers

A densely packed disordered bilayer was generated using a multistep protocol, which is described in detail in the Supporting Information. In short, the following steps were carried out: (i) HFBI monomers in coarse-grained (CG) representation were placed at random positions in the *x*–*y* plane, modeled with the MARTINI22 force field.^72,73^ The layer was compressed laterally in a simulation with a lateral pressure of 1200 bar and a temperature of 3600 K, while stabilizing the internal HFBI structures with elastic networks. (ii) The system was cooled to 300 K using a simulated annealing. (iii) The two resulting densely packed monolayers were combined to form a bilayer, and the CG system was equilibrated at constant volume. (iv) The CG models were backmapped to atomistic models with the Backward software.^74^ Because the secondary structure and certain side chain arrangements disagreed with the crystal structures after the CG-to-atomistic backmapping, we ran simulations with additional restraints to anneal the structure toward the correct crystallographic secondary structure. (v) After the addition of water and NaCl at 0.1 mol/L, the system was simulated for 100 ns at 300 K and 1 bar using the same parameters as described above.

### Permeability Calculations

The permeation of water molecules across a membrane can be described by the permeation events per time (Φ(*t*)) through a given surface area (*A*):
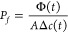
1where Δ*c*(*t*) is the concentration difference between the two sides of the membrane. Experimentally, water permeability is usually determined by the change in the volume of a pair of droplets (DIB) of different salt concentrations. Therefore, Φ can be described by the volume change of the droplets d*V*/d*t*·1/ν_W_ (ν_W_ = molar volume of water), and eq 1 results in^10,45,75^
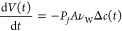
2By assuming a negligible salt concentration of the droplet of low salt concentration, eq 2 can be integrated over time to be able to use the values obtained from the experiments.
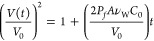
3*V*_0_ is the volume, and *C*_0_ is the concentration difference of the two droplets at the beginning of the measurement. Using this linear relationship, the permeability can be determined via the change in the volume of the droplets. The volume change is corrected for the volume loss of the droplets in the oil by assuming that the inflow into one droplet is equal to the outflow through the membrane of the second droplet. The volume change was investigated after the formation of a double layer until the double layer broke apart or a maximum time of 30 min was reached. Mean permeability values were then calculated by a linear fit.

In contrast to the experiments, the MD simulations contain an equal number of ions on both sides of the membrane due to the periodic boundary conditions. Therefore, there is approximately equal water flux in both directions, i.e., nearly zero net flux. However, the simulation system may be considered as an overlay of a water concentration gradient *c* in one direction with a gradient −*c* in the other direction, where *c* = 55 mol/L is the concentration of water. Hence, the number of permeation events *N* (in any direction) per simulation time *t* was translated into the membrane permeability with
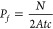
4Here, the factor 1/2 corrects for the fact that we summed permeation events in both directions. The water permeability *P*_*f*_ of the HFBI bilayers was computed as in Zocher et al.^76^ Accordingly, the position along the membrane normal (i.e., the *z* coordinate) was recorded for all of the oxygen atoms of water. Three layers were defined along the *z*-axis: a core layer spanning the membrane center and two outer layers that cover the water on top and below the membrane. A permeation event was registered if the particle passed from one outer layer through the core layer to the other outer layer. This protocol excludes the possibility that water molecules diffusing across the periodic boundaries are misinterpreted as permeation events. It is known that the TIP3P model produces diffusion coefficient values that are larger than the experimental observed ones.^77^ We therefore determined the diffusion coefficient of pure TIP3P water from a 5 ns simulation of pure water and found that the diffusion coefficient was increased by a factor of 2.49 relative to experiments at 298.15 K.^77^ Hence, we corrected the computed *P*_*f*_ by the same factor.

### Error Analysis in the Determination of Experimental Membrane Water Permeability

**Figure 7:**
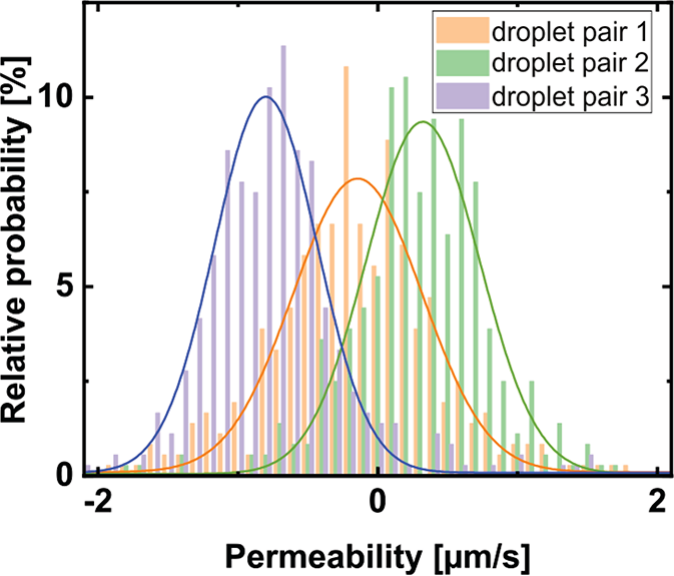
Distribution of permeability values calculated for every single time step for three pure HFBI wild-type membranes.

In Figure 7, the permeabilities for the pure HFBI membranes also assume negative values, which are physically implausible. After correction of the water loss into the hexadecane, the droplet with the higher salt content of the droplet pair does not always increase in volume, it can also shrink and thus lead to a negative sign of the permeability. Systematic errors, as e.g. in the calculation of the droplet volume from the cross-section images, might cause this observation; however, compared to the resolution of the experimental system, this systematic error is small: The permeability values scattered with ±0.5 μm/s around −0.4 μm/s (Figure 2b, data for pure HFBI). The resolution estimated from the inherent scatter in the droplet area determination is approximately 1 μm/s (standard deviation of the Gaussian distribution), as can be seen in the distribution of the evaluated permeability values for every time step (Figure 7). The two sources of error are therefore of the same order of magnitude, whereas the scattering determines the lowest possible resolution. Thus, in the current setup, the water permeability of HFBI bilayers is indistinguishable from zero.

## Supporting Information Available

Additional details to experiments and simulations (PDF)
