# Supplementary material for: Hydrophobin Bilayer as Water Impermeable Protein Membrane
Source: Langmuir. 2023 Sep 19;39(39):13790–800. doi: 10.1021/acs.langmuir.3c01006 (PMC10552762; doi:10.1021/acs.langmuir.3c01006)
Supplement: Supplementary file 1 — la3c01006_si_002.pdf [file la3c01006_si_002.pdf]

Supporting Information: Hydrophobin Bilayer as Water Impermeable Protein Membrane

Friederike Nolle,[†,‖] Leonhard J. Starke,[‡,‖] Alessandra Griffo,[†,¶,⊥] Michael Lienemann,[§] Karin Jacobs,[†,¶] Ralf Seemann,[†] Jean-Baptiste Fleury,[†] Jochen S. Hub,[*,‡] and Hendrik Hähl[*,†]

†*Department of Experimental Physics, Saarland University, D-66123 Saarbrücken, Germany*

‡*Department of Theoretical Physics, Saarland University, D-66123 Saarbrücken, Germany*

¶*Max Planck School, Matter to Life, Jahnstraße 29, 69120 Heidelberg, Germany*

§*VTT Technical Research Centre of Finland Ltd., Espoo 02150, Finland*

‖*These authors contributed equally to this work.*

⊥*Max Planck Institute for Medical Research Heidelberg, 69120 Heidelberg, Germany*

E-mail: jochen.hub@uni-saarland.de; h.haehl@physik.uni-saarland.de

Supporting Information

Water Transport Across a HFBI Monolayer in Hexadecane

The solubility of water in n-hexadecane is very low (ca. 3 mmol/L at 30 °C[1]), yet not zero. Therefore, water droplets in hexadecane will shrink over time as water molecules cross the water-oil interface and diffuse into the oil. To measure the water diffusion into hexadecane, single buffer droplets (with and without HFBI and KCl) were injected into a hexadecane bath at 30 °C with a hollow needle. Ensuring a total protein coverage of the droplet interface

for the droplets with HFBI, all droplets were added into hexadecane half an hour prior to measurement. Volume changes of these single droplets were observed with a top-view light microscope as described in the manuscript. To account for the loss of water into the surrounding oil, we quantify the volume of individual droplets consisting of pure water as well as of acetate buffer, with and without proteins in hexadecane over a period of 30 minutes. For all droplets, a clear decrease in volume was observed. The flux per area was thereby similar for all recorded droplets, *i.e.* independent of the presence of additional salt or proteins (Table S1).

Table S1: Water Flux and Diffusion Values of Different Solutions with and without Surrounding Hydrophobin Monolayer.

Solution	Water flux per area [10^{-4} µm/s]	Diffusion [10^3 µm^2/s]
Pure water	5.3 ± 1.1	5.5 ± 1.0
Buffer (I = 6 mM)	4.7 ± 0.9	4.8 ± 1.0
Buffer (I = 954 mM)	5.0 ± 1.0	5.1 ± 0.9
HFBI + buffer (I = 6 mM)	5.9 ± 0.8	6.0 ± 0.7
HFBI + buffer (I = 954 mM)	4.8 ± 0.7	5.1 ± 0.7

Using the diffusion equation and Fick's first law, we calculated the diffusion coefficient (D) of water from the drop into the oil by considering the solubility of water in hexadecane ($c_s = 0.003\,\mathrm{mol/L}$), for which we assume an approximately linear relationship with temperature,[1] *via*

$$D = \frac{V_0 - V(t)}{4\pi c_s \upsilon_w \int_0^t r(t)dt'} \tag{1}$$

where $\upsilon_w = 18\,\mathrm{g/mol}$ is the molar mass of water, V is the current droplet volume and V_0 is the droplet volume at time $t = 0$ and r is radius of the droplet. Complete saturation of the hexadecane with water was not achieved because the additional oil-air interface at the top of the oil bath allowed the water to evaporate. Effective diffusion values were determined to be in the range of 4–6·10^3 µm^2/s using eq. (1), which take a possible additional hindrance by an interfacial layer into account.

Thus, the volume change of droplets in the DIB setup is not solely caused by the water transport across the membrane that is formed between two droplets of different salt concentration, but also by the diffusion of the buffer through the monolayer of the drops in contact with the oil. In most studies[2–4] using the DIB method, this effect can be disregarded as the flux through a lipid membrane between two droplets is, typically, much larger than the self-dilution of the individual droplets. However, for the measurement of permeabilities in the µm/s range as relevant in our case, buffer diffusion into the oil must be taken into account.

a) b)

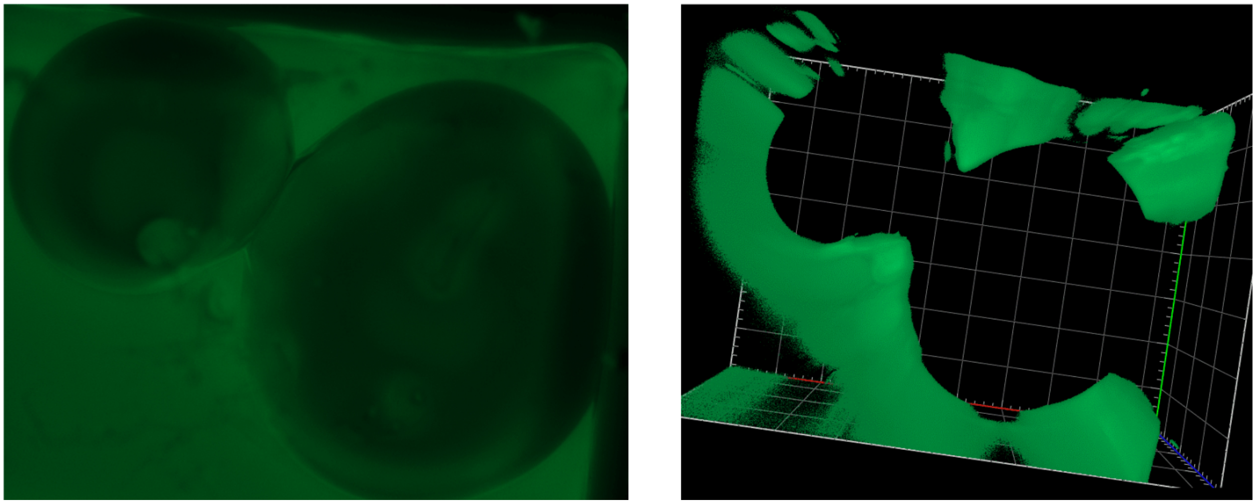

Figure S1: a) Top view of two identical HFBI-coated buffer droplets. Green shows the fluorescence signal of the BODIPY in the entire setup. b) 3D image stack in 5 µm steps over the z-height of the formed HFBI membrane. Green represents the fluorescence signal of the dyed oil in the plane of the membrane and excludes the dye above or below the membrane. A square of the drawn grid has a length of 600 µm each in x-, y- and z-direction.

Oil–Free Membrane

To visualize the oil around the droplets and investigate a suspected oil layer between the droplets (Figure S1), fluorescence microscopy measurements were performed with hexadecane stained with BODIPY (Thermo Fisher - 493/503 dye (Cat. No. D3922)). Preparation steps were identical to the permeability measurements described in the main text. Only the salinity

difference was chosen to be zero. A stack of microscopy images was recorded with height steps of 5 µm between the slices at an Axiovert 7 Zeiss with a colibri 7 (LED) illumination and rendered as a 3D image by software Zen Zeiss. In the images, no signal in the fluorescence channel could be detected in the contact area between the droplets. Thus, no evidence of oil between the individual HFBI layers was found, which could form an additional permeation barrier.

Data Processing

To extract the permeability from the droplets' volume change data, the volume loss into the surrounding oil was added to the data, as described in the main text. In Figure S2 data from the main text are presented after this correction step. Figure S2a depicts the situation for a pure HFBI bilayer showing almost no volume exchange through the bilayer and S2b the situation for a 0.4:1 HFBI-dCBM:HFBI WT ratio, showing a water flow through the membrane from the droplet of low salt concentration into the droplet of high salt concentration.

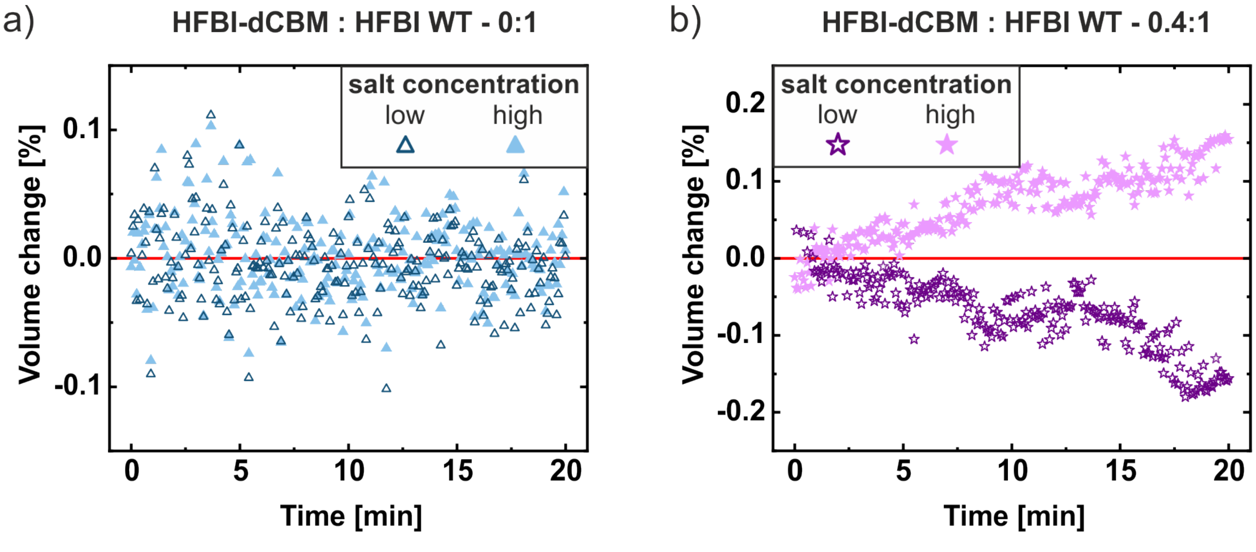

Figure S2: Relative volume changes, corrected for volume loss into oil, of two droplet pairs with different HFBI-dCBM:HFBI-WT ratios (a) 0:1 and b) 0.4:1). In each case, the filled symbols represent the droplet with high salt concentration and the open symbols represent the droplet with low salt concentration. The osmotic concentration difference was a) 1.717 osmol/L and b) 0.086 osmol/L. Temperature was kept constant at 30 °C.

Water Permeability Using NaCl as Osmolyte

Volume changes of two different droplet pairs with NaCl (1.717 osmol/L) instead of KCl as the osmotic pressure causing salt were recorded, resulting in permeabilities below the resolution limit ($P_f = 0.7$ and $0.4\,\mu m/s$) similar to droplets with KCl. Experimental setup was else identical to the measurements with KCl. Figure S3 compares measurements with NaCl and KCl.

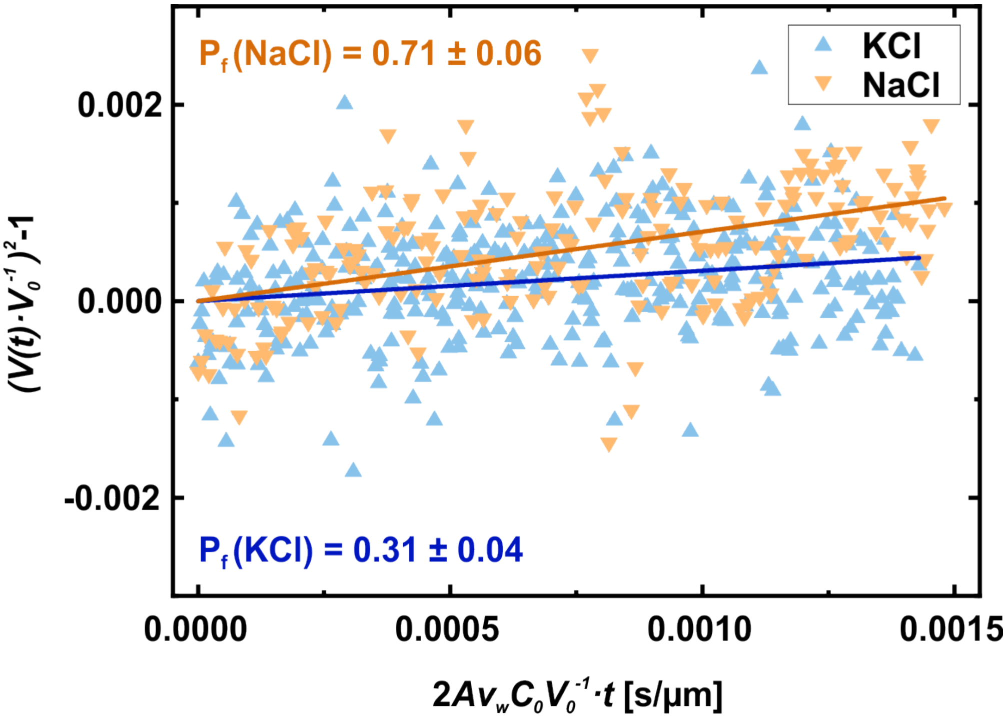

Figure S3: Comparing measurements using NaCl instead of KCl as the osmotic active molecule in the droplet of high salt concentration. The axes are chosen according to formula 3 so that the slope of the linear fit of the data corresponds to the mean permeability value of the membrane shown.However, both permeability values are below the resolution limit of our experiment.

MD Simulation of HFBI Monolayer at the Oil–Water Interface

We ran an additional simulation of the HFBI monolayer at an hexadecane–water interface to investigate the stability of the honeycomb structure under the experimental conditions present in the DIB setup. As starting configuration, we used the HFBI monolayer based

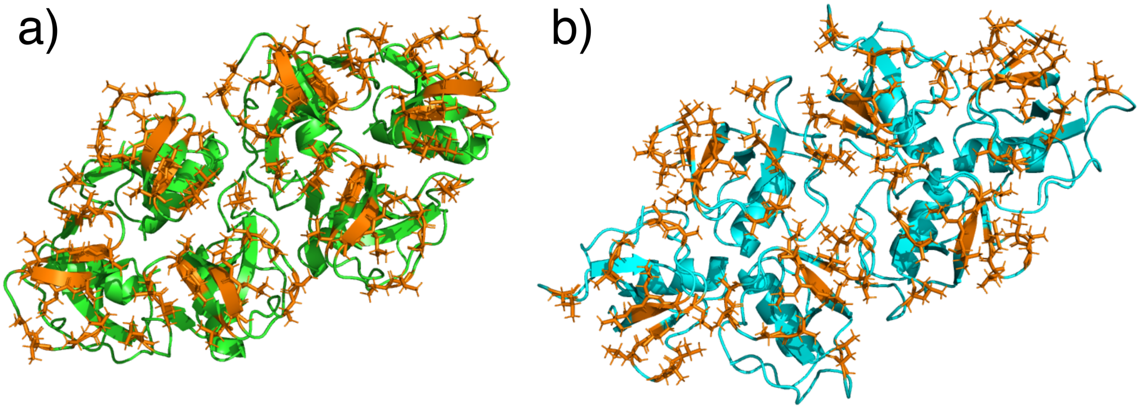

Figure S4: Proposed unit cells of HFBI hexamers from docking simulations[5] termed a) HFBI-α and b) HFBI-β. Monomers are shown in cartoon representation with the hydrophobic patch represented as orange sticks. Note the differences between the HFBI monomer interfaces as evident from the distinct orientation of the α-helices.

on the β unit cell at the air–water interface as described in the main text. An additional layer of hexadecane oil with a thickness of $\sim 3.2\,\mathrm{nm}$ was generated in a separate simulation box with identical x-y dimensions, composed of 1560 molecules corresponding to a density of $0.77\,\mathrm{g/cm}$ at room temperature.[6] This layer was inserted above the hydrophobic patch of the HFBI monolayer. Parameters for hexadecane were taken from alkane parameters by CHARMM36m, augmented with alkane–water interactions suggested by Krämer *et al.*.[7] Lennard-Jones interactions were treated with the cut-off scheme as described in the CHARMM specifications. The temperature was controlled at 310 K by velocity rescaling[8] and the pressure at 1 bar using the Berendsen barostat.[9] Flat bottom position restraints with a force constant of $200\,\mathrm{kJ/mol\,nm^2}$ for both the oil and the water layer were defined such that jumps over the periodic boundary were prevented while allowing the transport through the protein layer. We ran 10 ns for equilibration at constant volume, followed by 25 ns equilibration at constant pressure with pressure coupling only applied in x-y direction. Next, we carried out a 400 ns production simulation at 1 bar and 310 K. Water diffusion through the HFBI monolayer was detected using the method used to calculate water permeation through the bilayer (see main text). Here, the central layer was defined by the maximum and minimum z coordinate of the hydrophobic patch taken from the last simulation frame.

Consequently, the upper layer corresponded to the water phase and the lower layer to the oil phase.

During the simulation, water slowly penetrated the oil phase and vice versa, resulting in minor local deformations of the HFBI monolayer (Figure S5). Nevertheless, the honeycomb structure remained stable over the whole simulation. In the simulation time, we observed approx. 100 events of water molecule diffusion into the oil phase, showing that the simulations follow qualitatively the experiential observations. Quantitatively, however, we observed fewer events compared to simulations, which is expected because the TIP3P water models does not reproduce the experimental water/oil partition coefficient. This is explained by the fact that TIP3P has been parameterized to reproduce bulk properties and not properties of individual water molecules in oil, as has been reported previously.[10]

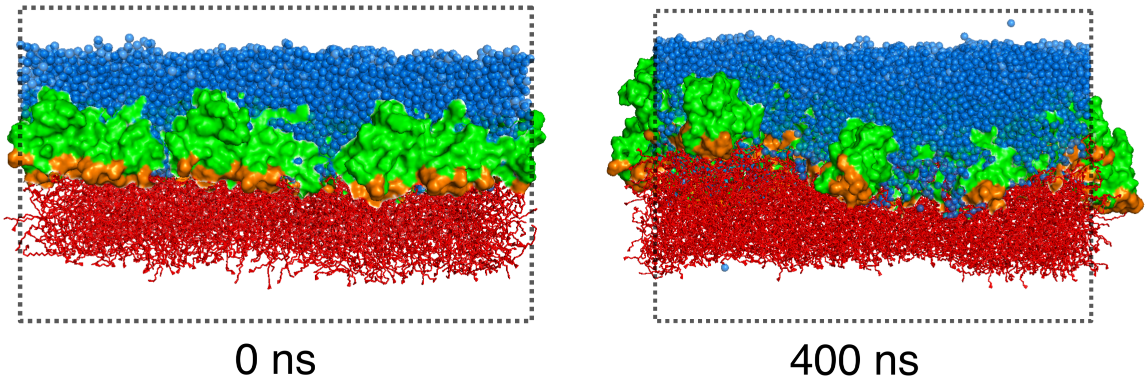

Figure S5: Snapshots of simulations of HFBI monolayer at the oil–water interface at 0 and 400 ns. The protein is shown in surface representation in green with the hydrophobic patch colored in orange. Hexadecane is shown as red sticks and water oxygens as blue spheres respectively. Dotted lines indicate the simulation box.

Variation of Force Fields and Water Model

To exclude that the choice of the protein force field or water influences the key findings of this study, we carried out additional simulations of the 'dense' HFBI bilayer based on the β unit cell with using the following combinations of protein force field and water model: AMBER99SB/TIP3P, OPLSaa/TIP3P, or CHARMM36m/OPC. For AMBER99SB/TIP3P,

Lennard-Jones interactions were truncated at 1.0 nm; for the other two combinations, Lennard-Jones interactions were truncated at 1.4 nm. All other simulations parameters were chosen as described above. Each setup was simulated at least for 15 ns and the water density profile was computed for different time intervals as shown in Figure S6. In all cases water penetrated the membrane core within few nanoseconds, demonstrating that the key findings are not influenced by the force field.

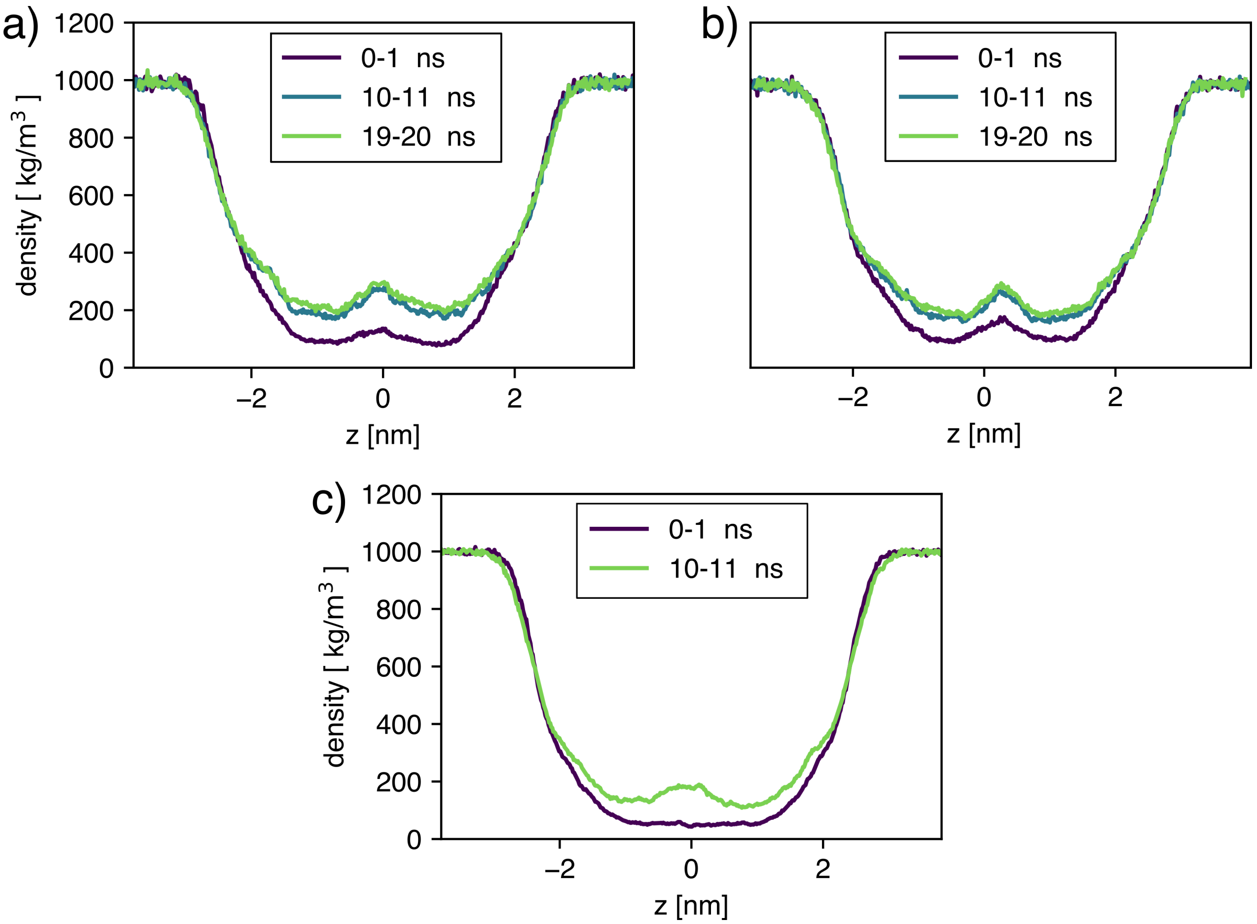

Figure S6: Water mass densities from simulations of the ‘dense’ HFBI bilayer based on the HFBI-β unit cell using different force fields and water models. Densities are averaged over intervals of 1 ns each. a) Simulation using the AMBER99SB force field and the TIP3P water model, b) OPLSaa force field and TIP3P water model, and c) CHARMM36m force field and the OPC water model. After 10 ns, water has penetrated the hydrophobic membrane core ($z \approx 0$ nm), independent of the choice of the force field or water model.

MD Simulations are Compatible with Oil-Free Hydrophobin Bilayers

To rationalize the low water permeability found experimentally, we hypothesized that a thin hexadecane layer of only a few atom layers remains between the monolayers and, thereby, prevents water transfer between neighboring hydrophobin monolayers. To test such an hypothesis, we carried out two MD simulations with a thin hexadecane film between the two hydrophobin layers with thicknesses of 1.3 nm and 0.5 nm, corresponding to approximately 6 or 3 hexadecane molecules per hydrophobin monomer respectively (Figure S7). The bilayer simulation system with the central hexadecane film was built from the dense bilayer configuration based on the HFBI-β unit cell. Again, each monolayer was composed of 3×3 unit cells. The protein bilayer was setup as described before, however with an additional gap in between the monolayers. The thin layer of hexadecane with a thickness of ~ 1.3 nm was generated in a separate simulation box with identical x-y dimensions, composed of 676 molecules corresponding to a density of 0.77 g/cm at room temperature.[6] The hexadecane layer was inserted into the gap between the HFBI monolayers, and the system was compressed in z-direction, while constraining the x-y-coordinates of the hexadecane atoms. To generate another system with an even thinner hexadecane layer, every second oil molecule was removed from the initial setup, followed again by compression along the z direction, thereby reducing the distance between the monolayers while maintaining the hexadecane film structure. The temperature was again controlled at 310 K with the velocity-rescale thermostat[8] and the pressure to 1 bar using the Parrinello-Rahman barostat.[11] For each system, one simulation of 400 ns was performed.

However, the hexadecane film ruptures within the simulation time and allows water leakage over the membrane. Simultaneously, the hexadecane aggregates to form oil droplets at the monolayer cavities. This effect was even more pronounced in the simulation with the reduced amount of hexadecane (Figure S7). In the experimental context, these oil droplets would eventually be absorbed by the oil reservoirs. These findings agree well with the notion

of an oil-free hydrophobin bilayer[12] and with the absence of BODIPY inside the membrane in the experiments described above (Figure S1). In addition, this data suggest that the low water permeability cannot be explained by residual oil but must instead be a consequence of tight packing between hydrophobin monomers.

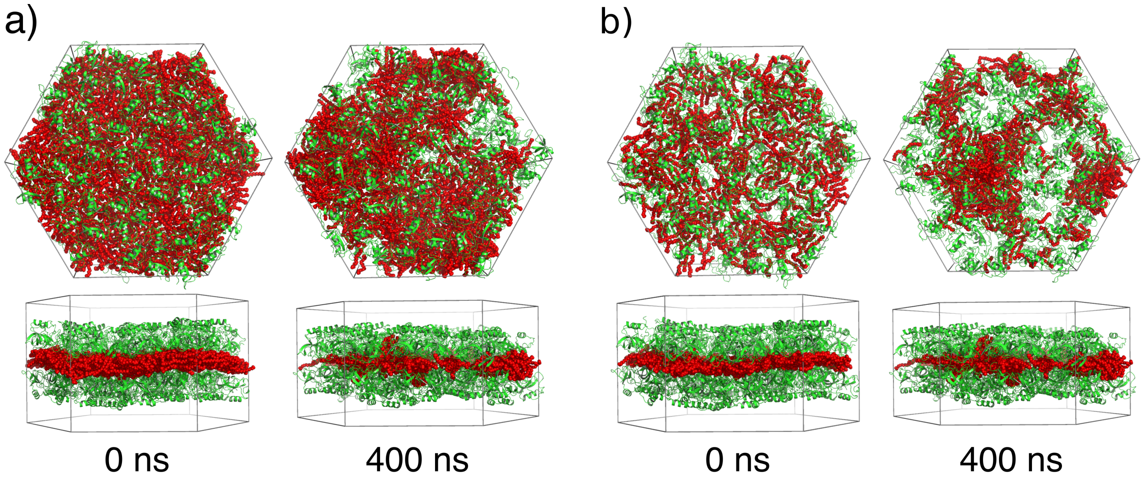

Figure S7: Simulation snapshots of simulations of HFBI bilayer with a hexadecane oil film (red) in between the protein layers with a) 6 or b) 3 hexadecane molecules per hydrophobin. The oil film ruptures and the hexadecane molecules start to fill the cavities between the proteins as seen in the lower panel (400 ns snapshots).

Detailed Protocol of Laterally Compressed Disordered Bilayer Setup

To generate a structural model of a densely packed HFBI bilayer the following simulation protocol was run consisting of five steps, as outlined in the main text and described in detail in the following:

Coarse Grained Layer Setup and Compression (i): A single HFBI protein was extracted from the crystal structure (PDB database entry 2fz6[13]), crystal water and zinc ions were removed. The atomic structure was converted into coarse-grained (CG) representation with the martinize.py script.[14,15] The protein was stabilized with an elastic bond network between the backbone beads and a set of manually defined distance constraints to prevent large

side chain rotations, which would otherwise disrupt the secondary structure after backmapping to atomistic resolution (Table S2). The force constants for these restraints were set to 500 kJ/(mol·nm^2). 50 monomers were placed in a plane at randomly selected x-y positions with the hydrophobic patches all facing towards the same direction, consistent with the orientation at an air–water interface. To rapidly compress the system, short simulations at $T = 3600$ K and $p = 1200$ bar were carried out for 20 ns. During those simulations position restraints in z-direction with a force constant of 1000 kJ/(mol·nm^2) were applied to ensure that the monolayer does not fold or bend. The time step was set to 1.25 fs to avoid instabilities during the simulation. Here, the cut-off values for Lennard-Jones and Coulomb interactions was set to 0.8 nm.

Annealing (ii): An annealing simulation was performed to relax the system and to allow the formation of tight and stable contacts between the monomers. To this end, the temperature was linearly decreased down to 300 K over a range of 100 ns.

Bilayer Setup and Constant Volume Equilibration (iii): The final frame of two independent compression simulations (steps i, ii) were equilibrated at constant volume for another 10 ns at 300 K. Afterwards both monolayers were overlayed, such that the hydrophobic cores are oriented inwards. The energy of the system was minimized, and the system was simulated for another 10 ns.

Backmapping to Atomistic Resolution (iv): Backmapping of the coarse grained systems followed the method by Wassenaar *et al.*[16] which consists of the actual backmapping process followed by a set of short MD simulations to relax the system. One potential issue of the backmapping procedure to an atomistic model is a potential loss of secondary structure. While certain deviations may be restored by running a long equilibration of the protein,[16] a full recovery of the secondary structure was only achieved by taking additional measures. Accordingly, we added (i) an additional elastic bond network between the C_α

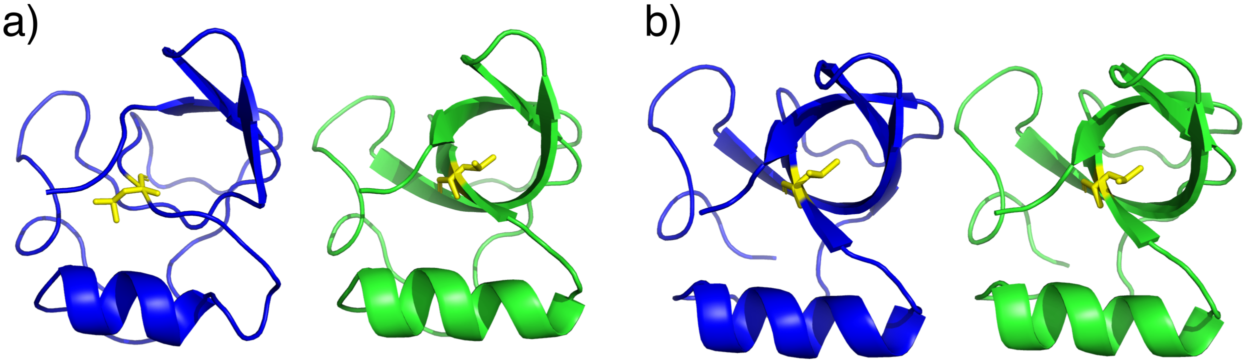

Figure S8: Comparison between a backmapped HFBI structure (blue) with the original crystal structure (green) before (a) and after the refinement of the secondary structure (b). An example for a side chain flip (LYS 56) is highlighted in yellow in each structure.

atoms and (ii) distance restraints between atoms to enforce the formation of the original hydrogen bond network of the crystal structure, which defined the secondary structure. For this purpose, the initial distances for the hydrogen bonds in the crystal structure were used. By gradually turning on these hydrogen bond restraints, the original secondary structure can be restored. First, a short pulling simulation was performed over 100 ps, with increasing the force constants from 40 to 4000 kJ/(mol nm^2), followed by a pulling simulation at constant force over 10 ns. Throughout the simulations, the elastic network force constants were kept at 500 kJ/(mol nm^2) as before. Visual inspection revealed excellent agreement of the refined HFBI structures with the crystal structure conformations (Figure S8). In the initial backmapped structure most of the characteristic beta barrel is lost, due to lost hydrogen bonds after large side chain rearrangements (Figure S8a). After the pulling simulations, the flipped side chain is in the correct orientation and the secondary structure of the backmapped protein is fully restored (Figure S8b).

Solvation (v): The refined dense bilayer was solvated with TIP3P water. Na^+ and Cl^- ions were added to a concentration of 0.1 mol/L followed by an energy minimization. The solvent structure was equilibrated at constant pressure conditions over 2 ns with position restraints on the backbone atoms. Subsequently, all position restraints were removed and

the system was simulated for 100 ns at 300 K and 1 bar.

Table S2: Additional distance restraints in the coarse-grained martini HFBI setup between beads i and j indicated by bead type, residue name, and residue number. Abbreviations of bead types: BB - backbone beads, SCk - k^{th} side chain bead

Bead i	Bead j	Distance [nm]
SC1 CYS 18	SC1 LEU 55	0.4
SC1 GLN 21	BB CYS 68	0.55
SC3 PHE 43	BB LEU 55	0.4
SC3 PHE 12	SC1 LEU 25	0.44
SC1 GLN 16	BB ALA 62	0.5
BB CYS 56	SC1 GLN 69	0.58
SC1 PRO 15	SC2 PHE 43	0.57

Extended Equilibration does not Prevent Water Leakage

Given the large forces present during compression and backmapping, we furthermore tested an extended equilibration protocol of the ‘dry’ compressed bilayer prior to solvation. To this end, we first carried out a constant volume equilibration over 5 ns of the backmapped and refined structure followed by a constant pressure equilibration with pressure coupling applied only in x-y direction over 400 ns. Next, we solvated the system as described above and carried out short equilibration at constant pressure over 5 ns with position restraints applied on the protein backbone atoms using a force constant of 1000 kJ/mol nm^2. Finally, a 200 ns production simulation at 1 bar and 300 K without position restraints was carried out. In this setup, we again observed water penetration on the nanosecond time scale as evident from water densities calculated at different time intervals (Figure S9).

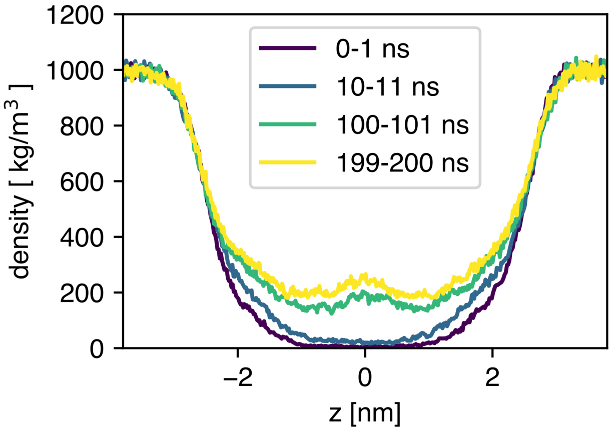

Figure S9: Water mass densities from simulations of the 'disordered' bilayer after a more extensive equilibration protocol which includes a prolonged 'dry' equilibration without water. Compared to the previous simulations (e.g. Figure 5) water penetration is slower but nevertheless reaches densities in the order of $\sim 200\,\mathrm{kg/m^3}$.

References

(1) Schatzberg, P. Solubilities of Water in Several Normal Alkanes from C7 to C161. *The Journal of Physical Chemistry* **1963**, *67*, 776–779.

(2) Milianta, P. J.; Muzzio, M.; Denver, J.; Cawley, G.; Lee, S. Water Permeability across Symmetric and Asymmetric Droplet Interface Bilayers: Interaction of Cholesterol Sulfate with DPhPC. *Langmuir* **2015**, *31*, 12187–12196.

(3) Michalak, Z.; Muzzio, M.; Milianta, P. J.; Giacomini, R.; Lee, S. Effect of Monoglyceride Structure and Cholesterol Content on Water Permeability of the Droplet Bilayer. *Langmuir* **2013**, *29*, 15919–15925.

(4) Fleury, J.-B. Enhanced Water Permeability across a Physiological Droplet Interface Bilayer Doped with Fullerenes. *RSC Advances* **2020**, *10*, 19686–19692.

(5) Magarkar, A.; Mele, N.; Abdel-Rahman, N.; Butcher, S.; Torkkeli, M.; Serimaa, R.; Paananen, A.; Linder, M.; Bunker, A. Hydrophobin Film Structure for HFBI and

HFBII and Mechanism for Accelerated Film Formation. *PLoS Computational Biology* **2014**, *10*, e1003745.

(6) DDB Saturated Lipid Density. `http://ddbonline.ddbst.de/DIPPR105DensityCalculation/DIPPR105CalculationCGI.exe?component=Hexadecane`, Accessed on May 25, 2023.

(7) Krämer, A.; Pickard, F. C.; Huang, J.; Venable, R. M.; Simmonett, A. C.; Reith, D.; Kirschner, K. N.; Pastor, R. W.; Brooks, B. R. Interactions of Water and Alkanes: Modifying Additive Force Fields to Account for Polarization Effects. *Journal of Chemical Theory and Computation* **2019**, *15*, 3854–3867.

(8) Bussi, G.; Donadio, D.; Parrinello, M. Canonical Sampling through Velocity Rescaling. *The Journal of Chemical Physics* **2007**, *126*, 014101.

(9) Berendsen, H. J. C.; Postma, J. P. M.; van Gunsteren, W. F.; DiNola, A.; Haak, J. R. Molecular Dynamics with Coupling to an External Bath. *The Journal of Chemical Physics* **1984**, *81*, 3684–3690.

(10) Venable, R. M.; Kramer, A.; Pastor, R. W. Molecular dynamics simulations of membrane permeability. *Chemical reviews* **2019**, *119*, 5954–5997.

(11) Parrinello, M.; Rahman, A. Polymorphic Transitions in Single Crystals: A New Molecular Dynamics Method. *Journal of Applied Physics* **1981**, *52*, 7182–7190.

(12) Hähl, H.; Vargas, J. N.; Griffo, A.; Laaksonen, P.; Szilvay, G.; Lienemann, M.; Jacobs, K.; Seemann, R.; Fleury, J.-B. Pure Protein Bilayers and Vesicles from Native Fungal Hydrophobins. *Advanced Materials* **2017**, *29*, 1602888.

(13) Hakanpää, J.; Szilvay, G. R.; Kaljunen, H.; Maksimainen, M.; Linder, M.; Rouvinen, J. Two Crystal Structures of Trichoderma Reesei Hydrophobin HFBI—The Structure of

a Protein Amphiphile with and without Detergent Interaction. *Protein Science* **2006**, *15*, 2129–2140.

(14) Marrink, S. J.; Risselada, H. J.; Yefimov, S.; Tieleman, D. P.; de Vries, A. H. The MARTINI Force Field: Coarse Grained Model for Biomolecular Simulations. *The Journal of Physical Chemistry B* **2007**, *111*, 7812–7824.

(15) Monticelli, L.; Kandasamy, S. K.; Periole, X.; Larson, R. G.; Tieleman, D. P.; Marrink, S.-J. The MARTINI Coarse-Grained Force Field: Extension to Proteins. *Journal of chemical theory and computation* **2008**, *4*, 819–834.

(16) Wassenaar, T. A.; Pluhackova, K.; Böckmann, R. A.; Marrink, S. J.; Tieleman, D. P. Going Backward: A Flexible Geometric Approach to Reverse Transformation from Coarse Grained to Atomistic Models. *Journal of Chemical Theory and Computation* **2014**, *10*, 676–690.
